# Ischemic Risk in Collodion Baby: An Orthopaedic Perspective

**DOI:** 10.1155/2020/1397465

**Published:** 2020-01-16

**Authors:** Pauline Besonhe, Pierre-Louis Docquier

**Affiliations:** ^1^Cliniques Universitaires Saint-Luc, Service d'Orthopédie et de Traumatologie de l'Appareil Locomoteur, Avenue Hippocrate 10, B-1200 Brussels, Belgium; ^2^Université Catholique de Louvain, Secteur des Sciences de la Santé, Institut de Recherche Expérimentale et Clinique, Neuro Musculo Skeletal Lab (NMSK), Avenue Mounier 53, B-1200 Brussels, Belgium

## Abstract

Collodion baby is a rare condition in which the baby is born surrounded by membranes called collodion membranes. The evolution of these membranes is towards cracking and peeling. Sometimes, retraction leads to hypoperfusion or ischemia (especially of fingers and toes). In case of acute ischemia, surgery is necessary. We report the case of a newborn in which surgery was necessary to free both fingers and toes from constrictive bands responsible for ischemia. In the absence of surgery, the constrictive bands can lead to amputation (pseudoainhum). The purpose of this case report is to expose the management and the role of an orthopaedic surgeon in the treatment of a collodion baby.

## 1. Introduction

The term “collodion baby” (CB) was first introduced by Hallopeau in 1884 [1]. It is not an entity as such but is an uncommon potential clinical presentation of several genetic disorders. In most cases, the cause is an autosomal recessive ichthyosiform disease: congenital reticular ichthyosiform erythroderma (OMIM number 609165), lamellar ichthyosis (146750), and harlequin ichthyosis (604777). Sometimes, the collodion baby phenotype can also be associated with Gaucher's disease (230800) and Sjogren-Larsson syndrome (270200). Other causes are rare and have been described in individual publication (Conradi-Hünermann-Happle syndrome (302960), neutral lipid storage disease (610717), ichthyosis vulgaris (146700), loricrin keratoderma (124500), trichothiodystrophy (601675),…) [2].

This phenotype is rare (1/50000 to 1/100000). The affected baby is covered by a thick, inelastic, and translucent membrane: “collodion membrane” [3]. This collodion membrane covers the entire surface of the body and results from a dysfunction in epidermal development. The skin is shiny and waxy, and the retractions can lead to ectropion, eclabium, lack of eyebrows, pilose thinning, and hypoplasia of nasal and auricular cartilage [3]. This pathology is quite serious with possible fatal risk (5%) caused by dehydration, electrolyte imbalance, sepsis, thermal instability, and respiratory and suction difficulties. The evolution of the membrane is oriented towards spontaneous and progressive shelling of the membrane within 3 or 4 weeks, preceded by the appearance of cracks. The evolution after the pickling is variable. The baby can heal spontaneously (10 to 20%) [4, 5] and shed membranes to reveal normal skin. Most often, the baby evolves in a more or less severe form of ichthyosis. In some cases, the baby has localized membranes in warm areas of the body (inguinal folds, scalp, and axillary hollow). This evolution is called “bathing suit ichthyosis.” It has been proven that this form of ichthyosis is due to mutations in the TGM1 gene, causing a unique enzymatic decrease in the “hot” areas of the body [6].

The collodion baby requires multidisciplinary care in a neonatal unit and may involve the orthopaedic surgeon. Indeed, during the peeling, the retraction of the band around the digit or toe can lead to a spontaneous amputation.

The transmission is autosomal recessive, and a prenatal diagnosis is possible. The potential serious nature of the pathology justifies that a prenatal diagnosis is suggested to families with at least one affected member. A diagnosis can be made from the twentieth week of amenorrhea by fibroscopy and feel skin biopsy. However, there is also an earlier diagnostic method that can be performed from the 10^th^ or 12^th^ week of pregnancy. This test is performed on material from chorionic villi by molecular diagnosis (PCR genomic). Many genetic mutations can be found in the baby collodion including TGM1, ALOXE3, ALOX12B, CYP4F22, PNPLA1, ABCA12, and NIPAL4. It is important to explore to determine the underlying form of ichthyosis, to provide prognostic elements, to allow genetic counselling, and to consider prenatal diagnosis in case of future pregnancy.

## 2. Case Presentation

It is a case of a girl born from nonconsanguineous parents, of Algerian origin. The Apgar score was 9/10/10 at 1 minute, 5 minutes, and 10 minutes.

**Figure 1:**
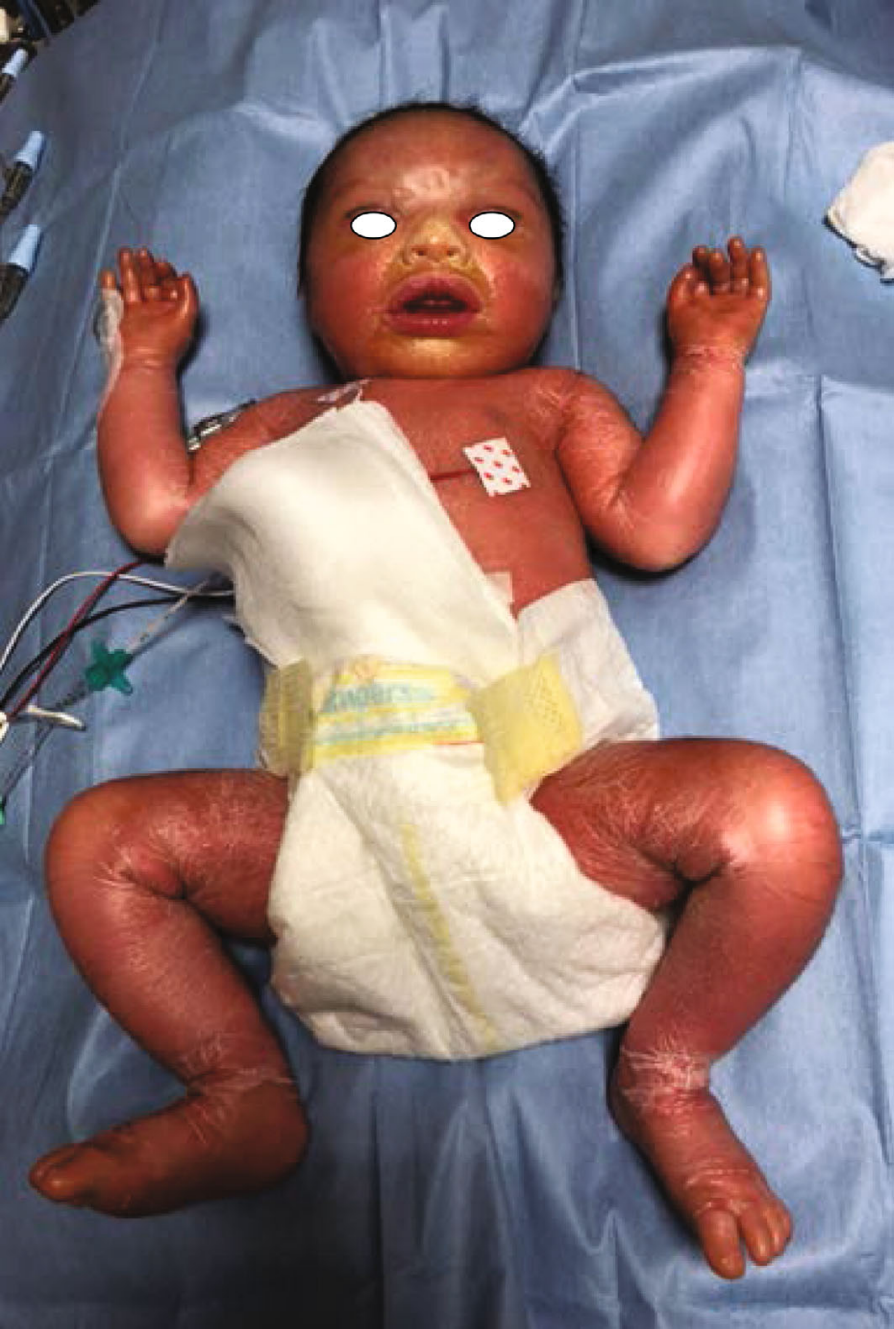
Collodion baby at birth.

She had several signs of baby collodion (Figure 1): bilateral ectropion, eclabium, stenosis of ear canals, and nasal vestibules. The whole body was covered with collodion membranes. She was the first child of the family, and there was a familial antecedent in a first-degree cousin of the baby who had the same presentation at birth.

**Figure 2:**
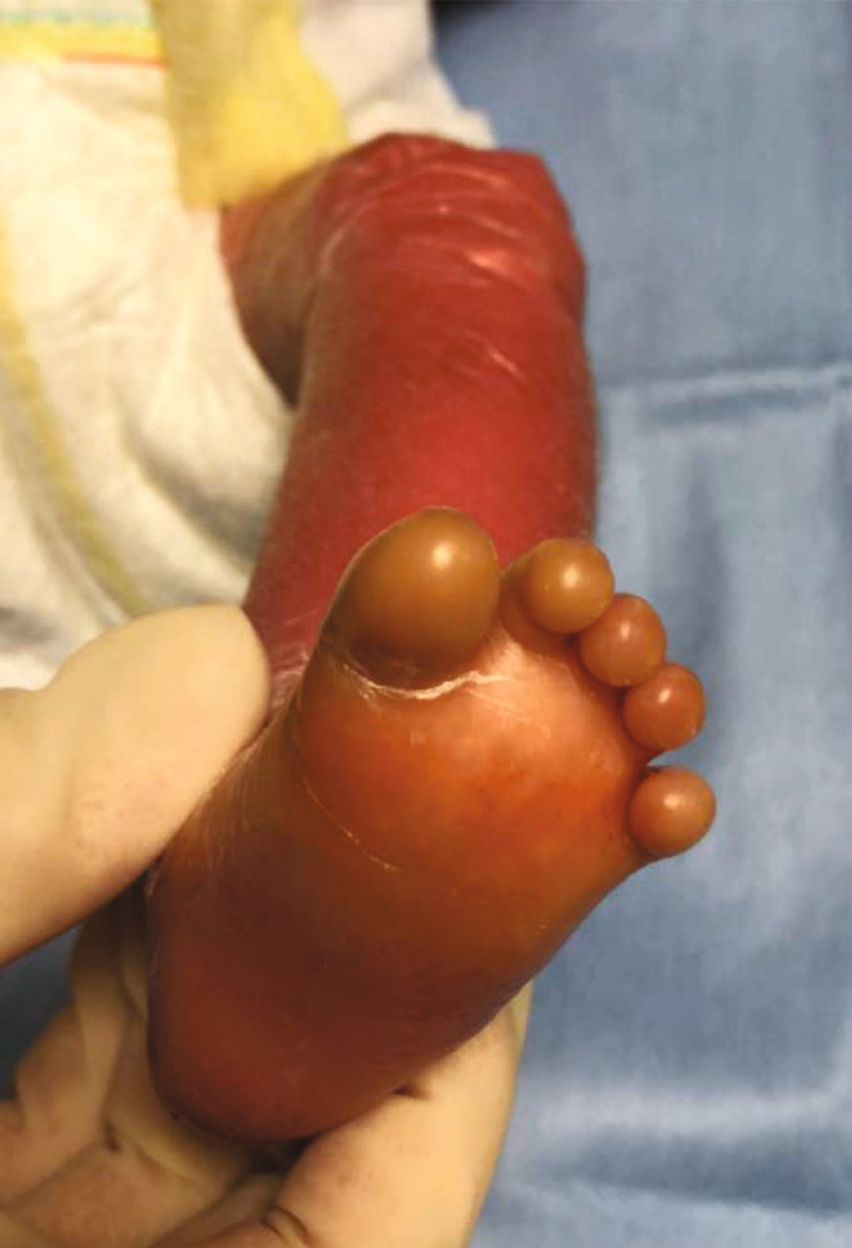
Collodion baby at birth: collodion membrane on the foot.

**Figure 3:**
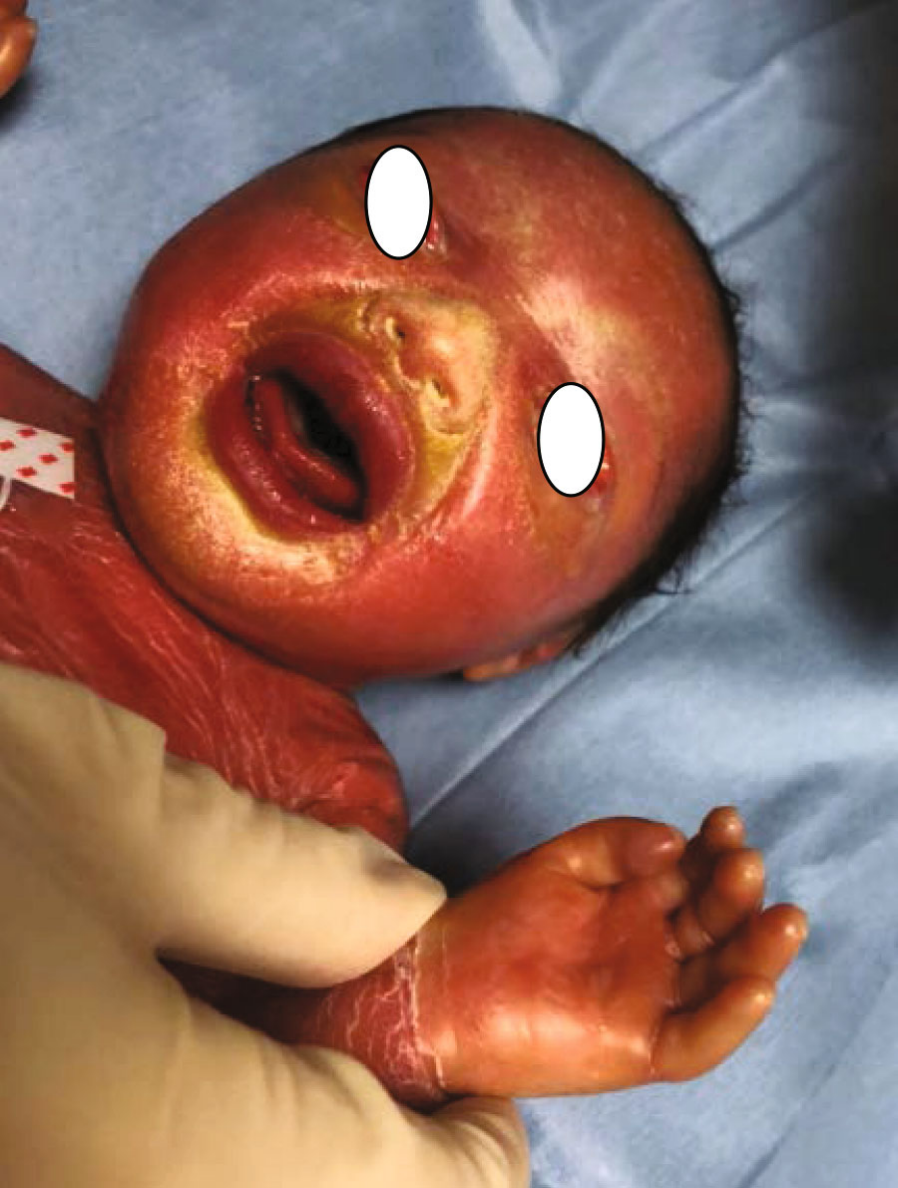
Collodion baby at birth: ectropion, collodion membrane on the hand.

The orthopaedic surgeon was called to give his opinion about the hypoperfusion of the fingers and of the toes. Clinical examination revealed that the hands and feet were covered with a flexible membrane without constrictive bands at that time (Figures 2 and 3). The hypoperfusion of the extremities was relieved by Vaseline massages.

**Figure 4:**
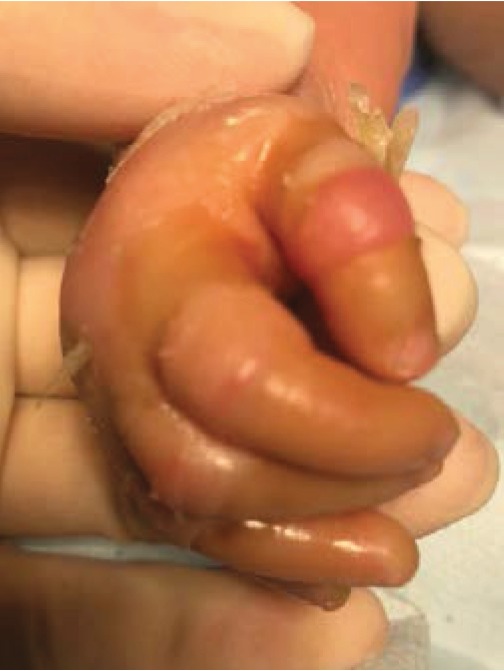
Constrictive bands on the fingers leading to ischemia.

**Figure 5:**
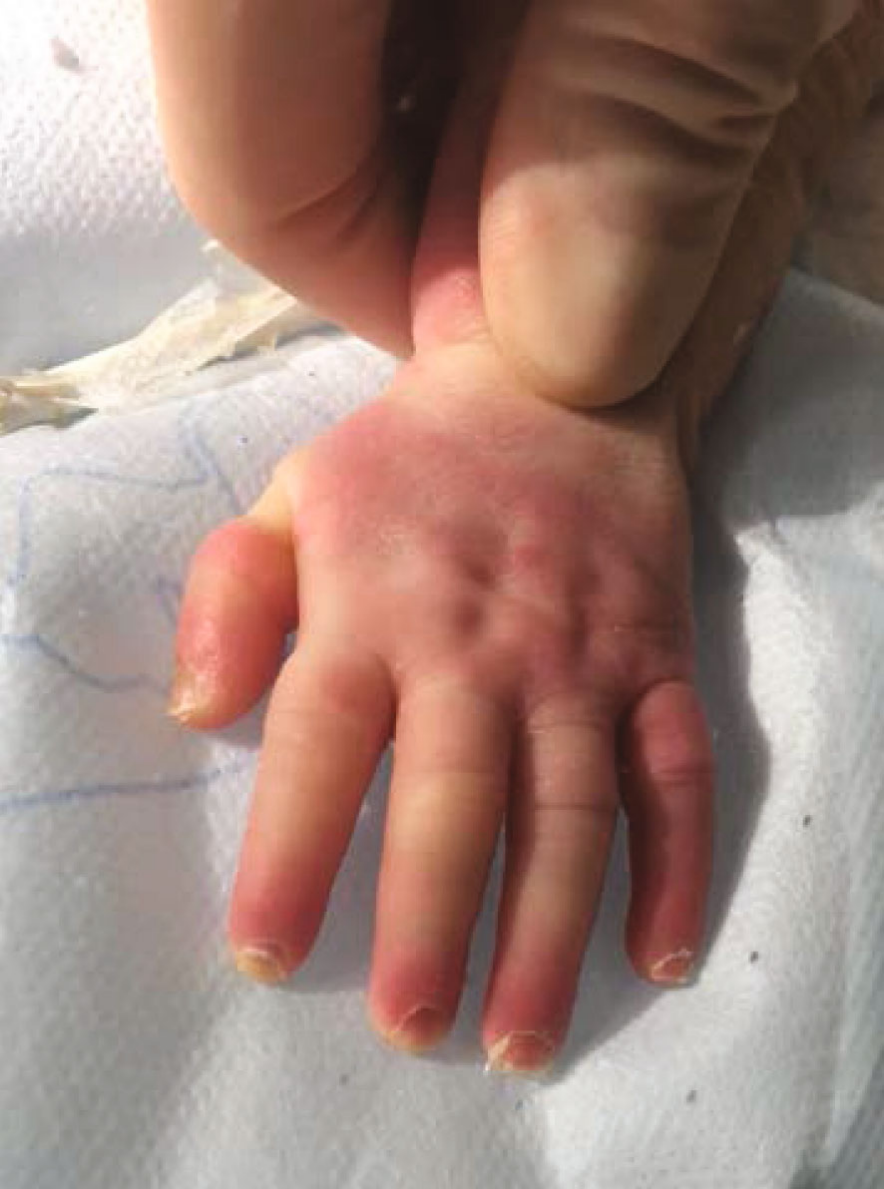
Hand after surgery.

**Figure 6:**
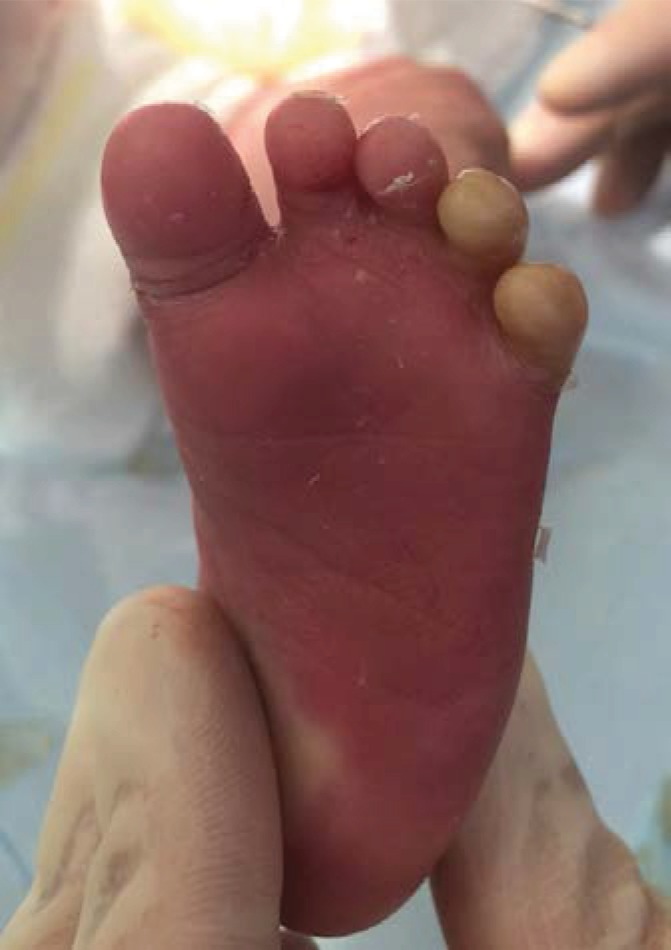
The foot during the surgery. See the difference before membrane resection (the last two toes) and after (the first three toes).

The newborn was admitted to the neonatology department and received specific care by a multidisciplinary team: dermatologist, ophthalmologist, neonatologist, ENT specialist, physiotherapist, and orthopaedist. The evolution was good; the little girl was able to close her mouth and eyes on day 7. The high humidity incubator was stopped on day 10. Unfortunately, on day 11, constrictive bands appeared at the root of the fingers (Figure 4) and of the toes causing ischemia. We decided to surgically remove this ischemia. Under general anaesthesia, the membrane was opened like a book and completely removed. All fingers and toes were released from the membrane. The underlying skin appeared intact (Figures 5 and 6). No other complications were subsequently observed.

After the 4-month follow-up, the child was fine. She presented only remnant thin membranes at the back, the scalp, the axillary hollows, and the inguinal folds. The rest of the skin looked healthy. This case corresponds therefore to a “bathing suit ichthyosis.” Genetic analyses revealed 2 mutations in the TGM1 gene. As mentioned earlier, this gene is involved in autosomal recessive congenital ichthyosis, especially in the “bathing suit ichthyosis form.”

## 3. Discussion

Pseudoainhum is a condition characterized by the appearance of a fibrous constrictive band around a limb. These constrictive bands can lead to self-amputation. Etiologies are diverse, and the baby collodion is one of the causes. Little is known about the physiopathology. Like in all the ichthyosiform diseases, the collodion membrane is caused by a problem in the formation, maintenance, and function of the stratum corneum of the skin. It is an epidermal cornification disorder.

There is no clear guideline for the treatment of the pseudoainhum. It can be medical or surgical. The literature does not recommend systematic antibiotic prophylaxis.

The first treatment is the lubrification and hydration of the skin. This treatment must be performed in order to maintain the skin's elasticity and to allow desquamation.

Medical treatment [7] is done by topical keratolytic. In some cases, good results are obtained 6 months after topical application of tazarotene twice a day. Some systemic treatments with etretinate or acitretin (retinoids) work well. However, these treatments cannot be tried in case of acute ischemia.

Surgical treatment consists of incision of the membrane without touching healthy skin. In general, the skin under the membrane is normal and the ischemia disappears instantly. No other care is required.

In conclusion, the collodion baby is a rare and serious disease. Care is multidisciplinary and may involve the orthopaedic surgeon. If collodion bands are constrictive, they must be cut and removed to stop ischemia.

These are the recommendations in the case of the collodion baby:

- Lubrication and humidification of the skin
- Monitoring until complete desquamation
- Going to the surgical procedure if there is ischemia
- Peeling the entire membrane
